# Development and application of a framework to estimate health care costs in China: The cervical cancer example

**DOI:** 10.1371/journal.pone.0222760

**Published:** 2019-10-01

**Authors:** Yi-Jun Liu, Adam Keane, Kate T. Simms, Jie-Bin Lew, Ju-Fang Shi, Carolyn Mazariego, Susan Yuill, Jose Jeronimo, You-Lin Qiao, Karen Canfell

**Affiliations:** 1 School of Public Health, Zunyi Medical University, Zunyi, China; 2 Cancer Research Division, Cancer Council New South Wales, Sydney, Australia; 3 Department of Epidemiology, Cancer Institute and Hospital, Chinese Academy of Medical Sciences and Peking Union Medical College, Beijing, China; 4 Global Coalition against Cervical Cancer, Arlington, Virginia, United States of America; 5 School of Public Health, University of Sydney, Sydney, Australia; 6 Prince of Wales Clinical School, Faculty of Medicine, UNSW, Sydney, Australia; Murdoch University, AUSTRALIA

## Abstract

**Objectives:**

Information on healthcare costs in low-and-middle-income countries is limited. This study presents a framework to perform healthcare cost estimates for each province in China.

**Methods:**

This study has two aims. Using cervical cancer as an example, the first aim is to use data (including micro-costing data) from one province to derive estimates for other provinces in China. This used provincial and national Chinese-language statistical reports and considered levels of service delivery, hospital-seeking behaviour, and the urban/rural population distribution. The second aim is to characterise the relationship between the reference costs estimated using the method mentioned above and two sets of cost estimates derived using simplified cost-scaling method with per capita Gross Domestic Product (GDP), and the Human Development Index (HDI). For simplified methods, regression modelling characterised the relationship between province-specific healthcare costs and macro-economic indicators, then we used the exponential fit to extrapolate costs.

**Results:**

Using the reference method, the estimated costs were found to vary substantially by urban/rural regions and between provinces; the ratios of highest to lowest provincial costs were 3.5 for visual inspection with acetic acid (VIA), 4.4 for cold knife conisation (CKC) and 4.6 for stage II cancer treatment. The HDI-based scaling method generally resulted in a better fit to reference costs than the GDP method.

**Conclusions:**

These reference costs for cervical cancer can inform cost-effectiveness evaluation of cervical screening and HPV vaccination in China. HDI-based methods for cost-scaling-based on social, as well as purely economic, factors-have potential to provide more accurate estimates.

## Introduction

Accurate estimation of healthcare costs plays an essential role in cost-effectiveness analysis. However, detailed information on the component costs of healthcare services for different health conditions is limited in many low and middle-income countries (LMIC). In practice, costs data used in the cost-effectiveness analyses conducted in LMIC settings are often extrapolated from micro-costing studies done in small-scale local studies or in other countries [1–3].

Cost-effectiveness analyses have been increasingly performed in mainland China over the past two decades. However, the numbers of these evaluations, which are essential for informing public health policy, are still limited in comparison to more developed settings. Furthermore, detailed disease-specific cost information for mainland China is limited. Extrapolating costs from one province to other provinces or to the national level is very complex due to large variations in population demographics, health care service models and funding in different regions. China has 31 provinces (not including Hong Kong, Macao and Taiwan) [4] and three basic administrative levels. The highest administrative level is provincial, followed by county, and township [5]. Residents in China can freely choose between township, county or provincial level hospitals for healthcare services (generally on a “user-pays” basis). The hospital-seeking behaviour varies between regions and between urban vs. rural, due in part to differences in the distribution of hospital locations and socioeconomic status [6]. Because health services costs increase for higher hospital levels, these differences in hospital-seeking behaviours are a key determinant of overall healthcare costs in different provinces.

A common practice in cost-effectiveness evaluations of cervical cancer prevention interventions in China has been to use data from a single hospital or local field study (or expert estimates), to represent the average cost at national level or at broad regional level such as rural or urban China [7–11]. One previous evaluation estimated cervical cancer screening, diagnosis, and treatment costs in the Chinese population based on the average hospital-seeking behaviour by income quintile and geographic location [12]. However, the hospital-seeking behaviour used in that study was based on assumptions, and detailed estimates for each province were not reported.

Micro-costing is a cost estimation method that allows for detailed assessment of the economic costs of health intervention in a particular setting [13]. Although PPPs are usually used to compare costs between different countries, when country-specific data are unavailable, the World Health Organization (WHO) has suggested that can be used to extrapolate unit costs from another setting to give an approximation of local costs [14]. Based on the analyses of the correlation between macroeconomic information and healthcare costs [15–17], several prior cervical cancer cost-effectiveness studies have used per capita GDP to scale country-specific hospital costs [1–3, 18]. However, scaling based on purely macroeconomic indices does not consider other socio-demographic factors that are likely to influence costs, including education level, distance to hospital facilities and transport infrastructure. The HDI is a composite measure of life expectancy, income and education level. The Chinese government has made significant improvements in the reduction of healthcare disparities over the past decade by introducing the national health insurance policy and increasing investment in education and healthcare [19]. Thus, HDI may be a better correlate of healthcare costs than per capita GDP or PPPs. To our knowledge, HDI has not been previously used to scale costs.

In this study we aimed firstly, to develop a framework using local data (including micro-costing data) to derive detailed reference healthcare cost estimates for each province in China; and secondly, to characterise the relationship between the costs derived via this detailed extrapolation method and those derived using simplified cost-scaling methods based on economic macro-indicators. This provides a verified simplified method applicable for other LMIC countries when country-specific data are unavailable or incomplete. We used the example of cervical cancer and considered costs associated with cervical cancer screening, diagnosis and treatment.

## Material and methods

### Derivation of reference costs (detailed costing methods)

A multistep process was used, which relied on analysis of provincial and national data from Chinese-language national reports including the 2018 China Health Statistics Yearbook [20], 2018 China Statistical Yearbook [21] and the 2013 5th National Health Service Survey [6]. The process was as follows. *Step 1*: Micro-costing data for cervical cancer screening, diagnosis, loop electrosurgical excision procedure (LEEP) and cold-knife conisation (CKC) from a county-level hospital, and cancer treatment cost from county and provincial level hospitals in Shanxi province in 2008, was adjusted to reflect 2018 prices in US$; *Step 2*: Equivalent costs for the same health care procedures, if conducted by hospitals at other levels in Shanxi (township or provincial), were derived via scaling each unit cost item in the micro-costing study according to whether it was incurred in the inpatient or outpatient setting, and also by general cost category (supplies, equipment, staff, drugs, or programmatic); *Step 3*: We extrapolated different hospital level costs from Shanxi to other provinces, based on average provincial variations for both inpatient and outpatient costs; *Step 4*: The distribution between urban and rural residents by province was determined, as was the distribution of care between the three hospital levels (hospital-seeking behaviour) at the region level; *Step 5*: Estimated aggregate costs for each procedure related to cervical cancer screening, diagnosis and treatment for each province were derived, taking into account all of the above factors; and *Step 6*: A weighted national average cost was derived, adjusting for the population in each province. Flow charts are present in S1 and S2 Figs. No line data was used, we only used published and publicly available data. A range of health care payers are involved in the costs included in the study. Health economic evaluations using these data would therefore represent the payer perspective.

#### Step 1. Processing of data from previous studies

A micro-costing study for cervical cancer screening, diagnosis, LEEP and CKC was performed in Xiangyuan county, Shanxi province, in 2008 [22–24]. The resources (supplies, equipment, staff and drugs) associated with clinic visits and laboratory testing, as well as programmatic costs were collected. Invasive cancer treatment costs, grouped by International Federation of Gynaecology and Obstetrics (FIGO) stage, were obtained from an audit of hospital charges in two hospitals: the Cancer Hospital of Shanxi Province (a provincial-level referral centre) and the Women and Children’s Hospital of Xiangyuan County, Shanxi Province (county level) [22]. For the current study, only direct medical costs were considered. All costs were converted to 2018 US dollars using the medical component of the consumer price index (CPI) [25]and exchange rate (1 US$ = 6.8632 CNY; 31 December 2018) [26]. The detailed original costs are listed in S1 and S2 Tables.

#### Step 2. Determination of the costs for each hospital level in Shanxi province

The micro-costing study was performed in a county-level hospital in Shanxi province. The county-level costs were scaled to provincial and township levels by applying separate multipliers to each category (supplies, equipment, staff, drugs and programmatic). The multipliers were derived from the 2018 China Health Statistics Yearbook [20], which reported the national average hospital staff expenditure, outpatient and inpatient costs differentials between the different hospital levels. Multipliers and the calculation process are described further in S3 and S4 Tables.

#### Step 3. Extrapolation to other provinces: Accounting for inpatient and outpatient cost differentials

Township/county/provincial level screening, diagnosis, LEEP and CKC costs, and county/provincial level cancer treatment costs, from Shanxi province were extrapolated to other provinces based on average provincial variations for both inpatient and outpatient costs. All screening, diagnosis tests and LEEP were assumed to be performed as an outpatient service, whereas CKC and cancer treatment were assumed to be inpatient services [27]. We assumed that outpatient services could be performed at all three hospital levels, but inpatient services could only be performed at county or provincial hospitals, consistent with the assumption used by the previous costing studies in China [22]. The province-specific hospital outpatient visit costs and hospital inpatient bed day costs were derived from the 2018 China Health Statistics Yearbook [20] and given in S5 Table.

#### Step 4. Determine the province-specific distribution by urban/rural and hospital-seeking behaviour

The urban proportion of the total population by province (S3 Fig) came from the 2018 China Statistical Yearbook [21]. Hospital-seeking behaviour (S6 Table) was considered separately for outpatient and inpatient service for residents, based on the 2013 5^th^ National Health Service Survey [6].

#### Step 5: Derivation of final costs for each province

The urban and rural proportions of the population along with the hospital-seeking behaviour can be used to obtain proportions visiting each hospital level. The resultant proportions for each province were multiplied by specific hospital level costs in each province to obtain a provincial weighted average cost for each procedure. For example, in Beijing, x% of residents are urban (step 4), and a%, b%, c% of these urban residents choose township, county and provincial level hospitals, respectively, for healthcare service; (100-x)% of Beijing residents are rural, and d%, e%, f% of these rural resident choose township, county and provincial level hospitals, respectively, for healthcare service (step 4). For a specific cervical cancer screening service, the costs were calculated as A, B, C in township, county, provincial level hospital (step 1–3). The equation for a provincial weighted average cost in Beijing is:

x%×(a%×A+b%×B+c%×C)+(100-x%)×(d%×A+e%×B+f%×C) (1)

#### Step 6: Derivation of national-level costs

A weighted national average cost was derived, adjusting for the population in each province using data from 2018 China Statistical Yearbook [21].

### Characterisation of the relationship between the reference costs and costs derived via simplified cost-scaling with two macro-indices

For this component, we performed the following steps: *Step 1*: We used regression modelling to characterise the fit between per capita GDP [21], HDI [28] and outpatient/inpatient cost for each province, the province-specific hospital outpatient visit costs and hospital inpatient bed day costs (for all conditions), derived from the 2018 China Health Statistics Yearbook [20]. *Step 2*: For any given province, we use an exponential fit to estimate the outpatient costs and inpatient costs for a particular cervical cancer component procedure as a function of their per capita GDP and HDI, applying the scaling algorithm to the costs in Shanxi Province (provincial average cost derived from the detailed reference method); and *Step 3*: we compared each of the component costs for each of the two simplified macro-indicator methods with those estimated using the reference method.

## Results

### Reference costs estimated from the detailed costing methods

Table 1 gives a summary of overall, urban, rural costs for cervical cancer screening, diagnosis and treatment costs estimated for national, eastern, middle, and western regions. Using the detailed reference method, the estimated population-weighted national overall costs was $2.9 for visual inspection with acetic acid (VIA), $9.4–11.3 for screening with Human papillomavirus (HPV) self-collection, $10.1–11.9 for clinical HPV screening, $5.0 for colposcopy, $7.6 for biopsy, $6.1 for endocervical curettage (ECC), $77.6 for LEEP, $280.7 for CKC and $1088.6, $1073.5, $1310.6, $1160.7 for invasive cervical cancer diagnosed at FIGO stages І, II, III and IV, respectively. Costs varied between regions and by urban vs. rural, for example, the ratio of eastern to western region costs for VIA was 1.2, for CKC 1.6, for FIGO stages II cervical cancer treatment 1.7; the ratio of urban to rural costs for VIA was 1.2, for CKC 1.4, for FIGO stages II cervical cancer treatment 1.5.

**Table 1:** Cost estimates for national and three regions using the detailed reference method (US$).

	National			Eastern region			Middle region			Western region		
	Overall	Urban	Rural	Overall	Urban	Rural	Overall	Urban	Rural	Overall	Urban	Rural
**Screening cost**												
VIA	2.9	3.1	2.6	3.3	3.5	2.8	2.7	2.9	2.4	2.6	2.8	2.4
VIA/VILI	3.5	3.8	3.0	3.9	4.2	3.3	3.2	3.5	2.9	3.1	3.4	2.8
careHPV*(self-sampling)	9.4	9.8	8.9	10.4	10.8	9.6	8.9	9.2	8.5	8.6	8.9	8.2
careHPV* (clinician-sampling)	10.1	10.5	9.3	11.2	11.6	10.1	9.5	9.9	8.9	9.1	9.6	8.7
HC2^#^(self-sampling)	11.3	11.7	10.6	12.4	12.9	11.5	10.6	11.0	10.1	10.2	10.6	9.8
HC2^#^ (clinician-sampling)	11.9	12.5	11.0	13.2	13.8	12.0	11.2	11.7	10.5	10.8	11.3	10.3
**Diagnosis cost**												
Colposcopy	5.0	5.4	4.5	5.6	6.0	4.9	4.7	5.0	4.3	4.6	4.9	4.2
Biopsy	7.6	8.2	6.6	8.5	9.2	7.1	7.0	7.5	6.3	6.8	7.4	6.2
ECC	6.1	6.5	5.3	6.8	7.3	5.7	5.6	6.0	5.0	5.5	5.9	5.0
**Pre-cancer treatment cost**												
LEEP	77.6	82.3	70.0	86.7	91.7	75.8	72.2	76.8	66.7	70.1	74.7	65.2
CKC	280.7	314.7	226.7	351.0	383.3	278.8	246.1	285.8	197.6	214.1	244.3	181.3
**Cancer treatment cost by FIGO**												
FIGO1	1088.6	1197.4	915.5	1354.3	1457.6	1123.0	957.1	1084.1	801.5	838.0	934.6	733.0
FIGO2	1073.5	1235.9	815.0	1352.4	1506.7	1007.0	937.6	1127.3	705.3	807.6	951.9	650.8
FIGO3	1310.6	1428.1	1123.7	1626.5	1738.0	1376.7	1153.9	1291.0	985.8	1013.5	1117.9	900.1
FIGO4	1160.7	1276.6	976.1	1444.0	1554.1	1197.4	1020.5	1155.9	854.6	893.5	996.5	781.6

*The careHPV assay (Qiagen, Gaithersburg, MD, USA); ^#^ The Hybrid Capture 2 (HC2) assay (Qiagen, Germantown, MD).

VIA, visual inspection with acetic acid; VILI, visual inspection with Lugol’s iodine; HPV, human papillomavirus; ECC, endocervical curettage; LEEP, loop electrosurgical excision procedure; CKC, cold knife conisation; FIGO, International Federation of Gynaecology and Obstetrics. Eastern region includes 11 provinces: Beijing, Tianjin, Hebei, Liaoning, Jiangsu, Shanghai, Zhejiang, Fujian, Guangdong, Hainan, Shandong; Middle region includes 8 provinces: Jilin, Heilongjiang, Shanxi, Henan, Anhui, Hubei, Hunan, Jiangxi; Western region includes 12 provinces: Inner Mongolia, Guangxi, Chongqing, Sichuan, Guizhou, Yunnan, Tibet, Shaanxi, Gansu, Qinghai, Ningxia, Xinjiang.

Costs varied substantially between provinces–for example, VIA costs in Beijing ($6.7) were more than twice that of the national overall ($2.9). The ratio of highest to lowest province average costs for VIA was 3.5, for CKC was 4.4, and for FIGO stages II cervical cancer treatment was 4.6. The detailed cost estimates for cervical cancer screening, diagnosis, pre-cancer treatment and cancer treatment for each province are shown in S7 Table.

### Characterisation of the relationship between the reference costs and those derived with the two macro-indices

**Fig 1:**
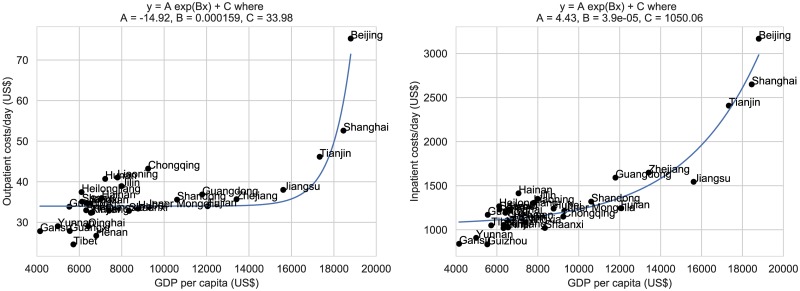
Exponential regression: Per capita GDP (Gross Domestic Product) as a predictor of hospital outpatient visit costs, hospital inpatient bed day costs.

**Fig 2:**
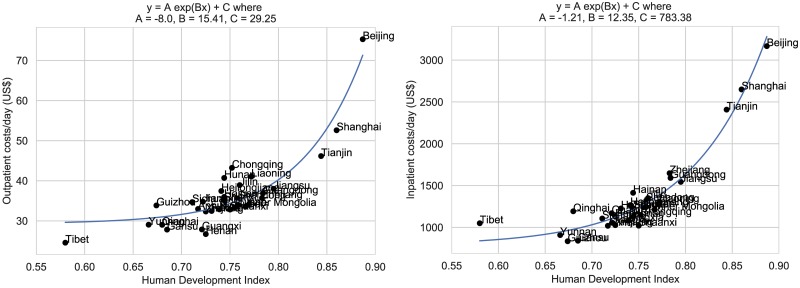
Exponential regression: HDI (Human Development Index) as a predictor of hospital outpatient visit costs, hospital inpatient bed day costs.

We used regression modelling to fit the province-specific hospital outpatient visit costs and hospital inpatient bed day costs to the per capita GDP and HDI for each province. Because the resulting distributions are skewed we found that they are better explained by exponential rather than linear fits. Fig 1 shows the province-specific GDP-based estimates for hospital outpatient visit costs and inpatient bed day costs. Fig 2 shows the province-specific HDI-based estimates for outpatient visit costs and inpatient bed day costs. In each case, the corresponding residual plots indicate that the exponential fit is reasonable (S4 and S5 Figs). For any given province, we use the exponential fit to estimate the outpatient costs and inpatient costs for a particular procedure as a function of their per capita GDP and HDI. Thus, to generate estimates of specific cervical cancer screening, diagnosis and treatment costs for other provinces, we took the outpatient/inpatient costs for Shanxi and applied the following multipliers:

$$\lambda_{\text{GDP}}=\frac{A_{GDP}\;exp(B_{GDP}*{per\;capita\;GDP}_{other\;province})+C_{GDP}}{A_{GDP}\;exp(B_{GDP}*{per\;capita\;GDP}_{shanxi})+C_{GDP}},$$ (2)

$$\lambda_{HDI}=\frac{A_{HDI}\;exp(B_{HDI}*{HDI}_{other\;province})+C_{HDI}}{A_{HDI}\;exp(B_{HDI}*{HDI}_{shanxi})+C_{HDI}},$$ (3)

where A_*GDP*_, B_*GDP*_, C_*GDP*_, A_*HDI*_, B_*HDI*_ and C_*HDI*_ are constants obtained from the exponential fit to provincial data on outpatient/inpatient costs and per capita GDP and HDI. Note that these constants, and consequently the multiplier, have different values for inpatient and outpatient costs.

In addition to HDI and GDP, we assessed whether other parameters could also be used to estimate costs across provinces. Parameters considered were household consumption [21], doctors per capita [21], hospital beds per capita [21], and proportion of hospitals (primary, secondary and tertiary)[20]. Out of each of these approaches, HDI was consistently the best parameter across all costs for both inpatient and outpatient procedures, with GDP being the next best (see S8 Table for detail). Combining HDI with these other measures tended to provide a slight benefit to outpatient estimates to the detriment of inpatient estimates. Only secondary hospital proportion and beds per capita added a slight benefit to both estimates although these were not statistically significant according to a t-test.

**Fig 3:**
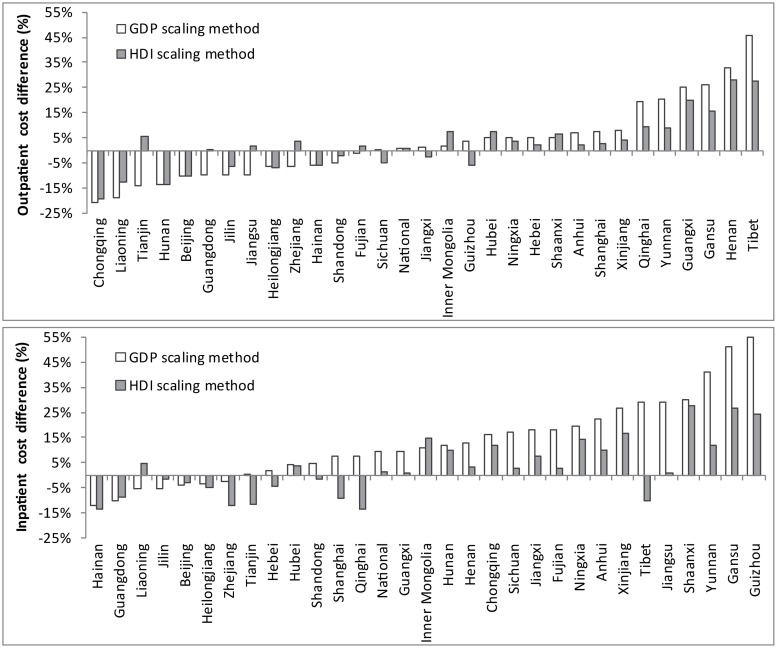
The average percentage difference between the reference costs and the cost estimated from the simplified methods.

Compared with the reference costs, average absolute difference (over province and procedure) ranged from 11.0–11.7% difference for outpatient costs and 15.3–16.7% different for inpatient costs whereas HDI-based scaling methods were consistently closer to the detailed methods estimate (7.9–8.4% different for outpatient and 8.9–10.0% different for inpatient). The average percentage difference varies by province (Fig 3). As a metric for whether the HDI-scaling method could be considered better than the GDP method in estimating inpatient costs, we calculated the absolute difference between both the HDI/GDP-scaled estimates and the detailed costs, averaged over all relevant procedures. This was repeated for each province and we then performed a t-test of the two sets of differences (for HDI and GDP over the provinces). We found for all inpatient tests that P<0.05, indicating a significant improvement of HDI over GDP; however outpatient tests were not significantly different to the HDI scaling method (P<0.20).

## Discussion

We presented a framework which considers the specific hospital levels and hospital-seeking behaviour in China in order to perform a detailed cost scaling exercise. This produced cost estimates for each province in China and for China nationally. To our knowledge, this is the first evaluation which comprehensively extrapolates costs across a large LMIC nation by taking into account detailed local factors. Overall, we found that costs vary substantially across provinces, with costs in Beijing, for example, being double the national overall. We also considered alternative simplified cost scaling methods using per capita GDP, which has been extensively used in prior studies, and validated this simplified approach against our more detailed scaled reference cost estimates. Finally, we proposed a third approach, which is also simplified, but based on HDI. We showed that this third method estimated costs more accurately for each province in China when compared to the simplified approach using per capita GDP.

We found that costs are generally higher in urban areas, which has also been noted in previous studies [7, 9, 10]. The estimated cost from Mo et al [8] was $427.2 for LEEP and $939.8 for CKC, which is substantially higher than our estimated national average level cost ($77.6 for LEEP, $280.7 for CKC). This may be because these costs were derived from one tertiary hospital (the highest level), whereas our cost is representative of all hospital levels over the whole country. Our estimated national overall cancer treatment cost for FIGO І-ІV was $1197–1428 for urban and $815–1124 for rural patients. Two earlier studies [7, 29] assessed cancer treatment costs based on hospital charges from a provincial hospital of Shenzhen (a large city in Guangdong province). Compared with our costs for urban Guangdong, $1474, $1524, $1758, $1572 for FIGO І, II, III, IV, their estimates of $1665, $2306, $2237, $2181 in Shenzhen, respectively, were higher. However, Shenzhen is the richest city in Guangdong province with $26156 per capita GDP in 2017 [30], which is 2.2 times higher than the average of Guangdong province ($11725) [31], and nearly 1.5 times higher than Beijing ($18795) [32]. Another study surveyed 6 rural and 12 urban clinical experts from 8 provinces in different regions of China to assess treatment costs for cervical cancer according to the service prices [9], and obtained a weighted value of $4247 per capita for both urban and rural populations, which is substantially higher than our national average cost and higher than the previous studies estimates. The differences may be due to the following factors: 1) the cost estimated from our study was adjusted for hospital-seeking behaviour and cost differences between township/county/provincial level hospital, whereas the other study did not consider differences in hospital seeking behaviour and hospital level [9]; 2) the cancer treatment cost in our study was not micro-costed but rather was estimated from medical charges reported in an earlier study from Shanxi; 3) only urban cancer treatment costs were considered in the prior study as they assumed that all the patients with diagnostically confirmed cancer would seek treatment in an urban area. Finally, a systematic review on the economic burden of cancer in China showed that the per capita direct medical cost for cervical cancer was very stable during the period 1996–2014 with a mean value 10 000 yuan (2018 US $1457) [33], which is similar to the costs we have generated across most provinces. Therefore, although the cost of treatment varies significantly between our estimates and these studies, it is important to note that the differences among the above studies themselves are also large. The national overall direct medical cost in a prior study [12] was $2.62 for VIA, $5.16-$5.77 for colposcopy, $6.92-$7.09 for biopsy and $77.73-$83.61 for LEEP, which was broadly consistent with our estimate: $2.9 (VIA), $5.0 (colposcopy), $7.6 (biopsy) and $77.6 (LEEP). Taking all of this together, our more detailed estimates are broadly consistent with previous estimates, but we have captured more detail on the heterogeneity between provinces.

Our findings suggest that scaling up cost across settings based on HDI provide estimates similar to the reference cost, whereas per capita GDP estimates tended to be higher than the reference cost. Furthermore, the HDI appeared to be a better match than a variety of other estimates too, such as those based on number of primary, secondary or tertiary hospitals; adding these macro-indices to an HDI-based regression did not appear to provide any additional explanatory power. This is likely because HDI captures other important factors, including education level and life expectancy, and some of these may influence hospital-seeking behaviour and other factors important for overall costs at the population level.

Over the past two decades, there has been considerable debate about whether GDP is a good measure of a country’s overall well-being. Some experts suggest that multi-dimensional indicators of development like HDI are preferable to measures of GDP growth [34]. WHO guidelines suggest that while PPPs are usually used to compare costs between different countries, they may also be used to extrapolate costs between settings when unit costs are not available for a local setting [14]. However, we could not use this method in the current study as there are no PPPs at a provincial level in China. Prior work has demonstrated that converting raw cost data into a percentage of per capita GDP for individual countries is a feasible approach to extrapolating direct medical costs across countries [35]. A local analysis from China, which compared medical service data between 20 provinces, suggested that there was no discernible correlation between medical service prices and the level of regional economic development [36]. This indicates that caution is needed when using GDP-based methods in China, and our study shows that HDI may be a better option.

Special attention should be given to Beijing and Tibet when extrapolating costs where healthcare costs are substantially different to the national cost average. The simplified scaling methods that used HDI and per capita GDP were less accurate for these regions. Along with the three administrative levels in China, hospitals in China are classified into primary/secondary/tertiary (the highest level), according to their bed size and the medical services they offer [37]. Beijing has the highest proportion (36%) of tertiary hospitals in China, which is 3.6 times larger than Tibet [20]. This likely influences the high costs in Beijing. Scaling up each cost component based on cost differentials in primary/secondary/tertiary hospital may be a good solution if adequate data is available. Other local factors may affect costing estimates for Beijing and Tibet, and care must be exercised when considering estimates for these provinces.

The framework we have provided can be adapted to the range of data available, with the caveat that simplifications may cause more divergence in the estimates. For the macro-index approach, a fitting function may still be used when only some data points are available. As described earlier, other indices such as household consumption, doctors per capita, or number of hospitals may be used as a proxy in the absence of HDI or GDP, although HDI appears to be most accurate in this study. For the detailed method, some countries may not provide a breakdown of hospital levels, rural/urban breakdown, or inpatient/outpatient components (such as drugs or surgery), but in each of these cases, one could use overall values. If health-seeking behaviour is not provided at the level of individual regions (provinces in our case), one may, for example, use the national average or a representative region.

One of the limitations of our study is that we have scaled costs based on one micro-costing study, namely Xiangyuan county of Shanxi province, and there are no other micro-costing data available for validation. However, we used a detailed multistep method to estimate reference costs which considered differentials between hospital levels, hospital-seeking behaviour, and the urban/rural population distribution in each province. This level of detail in deriving costs has not been previously performed for China and the same framework could be used to extrapolate costs for other cancers and other health conditions in China and in other LMIC countries. The other limitation is that we estimated cost from a payer perspective and would need to draw on or build the evidence base for additional data for out of pocket and indirect costs if a societal viewpoint was desired for the evaluation.

Due to the limited availability of cost data, most of the previous cost-effectiveness analyses of health care interventions divide China into urban and rural populations [7, 9, 10, 29], and some assumed that costs observed in, or estimated for, one region of China were nationally representative [8, 38]. Our study found that the cost of cervical cancer screening, diagnosis, and treatment varies between provinces and decreases from eastern to western regions. This is consistent with economic development patterns in China. Costs for HPV screening, biopsy, CKC and cancer treatment in rural eastern regions were higher than urban western regions. This indicates that a simple urban-rural division may not adequately capture the considerable heterogeneity of healthcare costs in China. The findings of this study will aid future cost-effectiveness analyses on cervical cancer screening and treatment related intervention in China by providing detailed and region-specific cost estimates.

## Conclusions

The costs of healthcare in China vary substantially not only between urban and rural populations but also between provinces. Our reference costs for cervical cancer provide important data to inform future cost-effectiveness evaluation of cervical screening and HPV vaccination in China. The comparison of the reference costs with those derived using macro-indicators suggests that HDI-based estimation methods for scaling costs have the potential to provide more accurate estimates that take into account social, as well as economic, determinants of healthcare costs.

## Supporting information

S1 Fig The flow charts for the detailed method and the simplified method. (PDF)

S2 Fig The detail process of step 2–3 (using a specific example of biopsies). (PDF)

S3 Fig The urban percentage of the total population by province. (PDF)

S4 Fig Residual plots of outpatient and inpatient costs for the GDP-based exponential fit. (PDF)

S5 Fig Residual plots of outpatient and inpatient costs for the HDI-based exponential fit. (PDF)

S1 Table Direct medical costs for cervical screening, diagnosis and pre-cancer treatment from micro-costing study (2018 US$). (DOCX)

S2 Table Direct medical costs for cervical cancer treatment from previous study (2018 US$). (DOCX)

S3 Table Multipliers used to extrapolate county-level cost to township and provincial level in Shanxi province. (DOCX)

S4 Table National average cost by hospital level based on data from the 2018 China Health Statistics Yearbook (partial data) (2018 US$). (DOCX)

S5 Table Province-specific hospital outpatient visit costs and hospital inpatient bed day costs (2018 US$). (DOCX)

S6 Table Hospital-seeking behaviour by urban/rural and region (%). (DOCX)

S7 Table Direct medical costs for screening, diagnosis, pre-cancer, and cancer treatment by province in China (2018 US$). (XLSX)

S8 Table Percentage difference of various fitting functions and data. (DOCX)

## References

[1] Campos NG, Kim JJ, Castle PE, Ortendahl JD, O'Shea M, Diaz M, et al Health and economic impact of HPV 16/18 vaccination and cervical cancer screening in Eastern Africa. International journal of cancer. 2012;130(11):2672–84. Epub 2011/07/01. 10.1002/ijc.26269 21717458 PMC3314721

[2] Goldie SJ, Kim JJ, Kobus K, Goldhaber-Fiebert JD, Salomon J, O'Shea M K, et al Cost-effectiveness of HPV 16, 18 vaccination in Brazil. Vaccine. 2007;25(33):6257–70. Epub 2007/07/04. 10.1016/j.vaccine.2007.05.058 . 17606315

[3] Campos NG, Castle PE, Wright TC Jr., Kim JJ. Cervical cancer screening in low-resource settings: A cost-effectiveness framework for valuing tradeoffs between test performance and program coverage. International journal of cancer. 2015;137(9):2208–19. Epub 2015/05/07. 10.1002/ijc.29594 25943074 PMC4910518

[4] The Central People’s Government of China. Administrative divisions of the People’s Republic of China 2013. http://www.gov.cn/test/2005-06/15/content_18253.htm.

[5] The Central People’s Government of China. Outline of China’s Administrative Division 2009. http://www.gov.cn/test/2009-04/17/content_1288030.htm.

[6] Center for Health Statistics and Information NHFPC. An Analysis Report of National Health Services Survey in China, 2013. Peking Union Medical College Press; 2015.

[7] Xu H, Zhao FH, Gao XH, Hu SY, Chen JF, Liu ZH, et al [Cost-effectiveness analysis on the once-in-a-lifetime cervical cancer screening program for women living in rural and urban areas of China]. Zhonghua liu xing bing xue za zhi = Zhonghua liuxingbingxue zazhi. 2013;34(4):399–403. Epub 2013/08/14. . 23937850

[8] Mo X, Gai Tobe R, Wang L, Liu X, Wu B, Luo H, et al Cost-effectiveness analysis of different types of human papillomavirus vaccination combined with a cervical cancer screening program in mainland China. BMC infectious diseases. 2017;17(1):502 Epub 2017/07/20. 10.1186/s12879-017-2592-5 28720082 PMC5516327

[9] Zhang Q, Liu YJ, Hu SY, Zhao FH. Estimating long-term clinical effectiveness and cost-effectiveness of HPV 16/18 vaccine in China. BMC cancer. 2016;16(1):848 Epub 2016/11/07. 10.1186/s12885-016-2893-x 27814703 PMC5097411

[10] Liu YJ, Zhang Q, Hu SY, Zhao FH. Effect of vaccination age on cost-effectiveness of human papillomavirus vaccination against cervical cancer in China. BMC cancer. 2016;16:164 Epub 2016/02/28. 10.1186/s12885-016-2207-3 26919850 PMC4768405

[11] Canfell K, Shi JF, Lew JB, Walker R, Zhao FH, Simonella L, et al Prevention of cervical cancer in rural China: evaluation of HPV vaccination and primary HPV screening strategies. Vaccine. 2011;29(13):2487–94. Epub 2011/01/08. 10.1016/j.vaccine.2010.12.085 . 21211586

[12] Levin CE, Sharma M, Olson Z, Verguet S, Shi JF, Wang SM, et al An extended cost-effectiveness analysis of publicly financed HPV vaccination to prevent cervical cancer in China. Vaccine. 2015;33(24):2830–41. Epub 2015/03/17. 10.1016/j.vaccine.2015.02.052 . 25770785

[13] Xu X, Grossetta Nardini HK, Ruger JP. Micro-costing studies in the health and medical literature: protocol for a systematic review. Systematic reviews. 2014;3:47 Epub 2014/06/03. 10.1186/2046-4053-3-47 24887208 PMC4036677

[14] World Health Organization. Making Choices in Health: WHO Guide to Cost-Effectiveness Analysis 2003. https://apps.who.int/iris/bitstream/handle/10665/42699/9241546018.pdf;jsessionid=3356F4CEF6F855295560B4F32AF2B555?sequence=1.

[15] Ke X, Saksena P, Holly A. The determinants of health expenditure: a country-level panel data analysis. World Health Organization. 2011.

[16] Piabuo SM, Tieguhong JC. Health expenditure and economic growth—a review of the literature and an analysis between the economic community for central African states (CEMAC) and selected African countries. Health economics review. 2017;7(1):23 Epub 2017/06/09. 10.1186/s13561-017-0159-1 28593509 PMC5462666

[17] Fuchs VR. The gross domestic product and health care spending. The New England journal of medicine. 2013;369(2):107–9. Epub 2013/05/24. 10.1056/NEJMp1305298 . 23697470

[18] Goldie SJ, Diaz M, Kim S-Y, Levin CE, Van Minh H, Kim JJ. Mathematical models of cervical cancer prevention in the Asia Pacific region. Vaccine. 2008;26:M17–M29. 10.1016/j.vaccine.2008.06.018 18945411

[19] Fang H. The Chinese Health Care System. http://international.commonwealthfund.org/countries/china/.

[20] National Health and Family Planning Commission of PRC. China Health Statistics Yearbook 2018: Peking Union Medical College Press; 2018.

[21] National Bureau of Statistics of China. China Stastistical Yearbook 2018: China Statistics Press; 2018.

[22] Shi JF, Canfell K, Lew JB, Zhao FH, Legood R, Ning Y, et al Evaluation of primary HPV-DNA testing in relation to visual inspection methods for cervical cancer screening in rural China: an epidemiologic and cost-effectiveness modelling study. BMC cancer. 2011;11:239 Epub 2011/06/15. 10.1186/1471-2407-11-239 21668946 PMC3141766

[23] Shi JF, Chen JF, Canfell K, Feng XX, Ma JF, Zhang YZ, et al Estimation of the costs of cervical cancer screening, diagnosis and treatment in rural Shanxi Province, China: a micro-costing study. BMC health services research. 2012;12:123 Epub 2012/05/26. 10.1186/1472-6963-12-123 22624619 PMC3461448

[24] Shi JF. Cost-effectiveness on Various Modalities of Cervical Cancer Screening in Rural China: Chinese academy of medical sciences & Peking union medical college; 2009.

[25] National Bureau of Statistics of China. Consumer Price Indices. http://data.stats.gov.cn/search.htm?s=CPI.

[26] Bank of China. Exchange Rate. http://www.boc.cn/sourcedb/whpj/enindex2.htm.

[27] University of Colorado. Conization and LEEP Treatments. https://cancer.coloradowomenshealth.com/cancer-treatments/surgery/conization-leep/index.html.

[28] Global Data Lab. Subnational Human Development Index (SD-2019). https://globaldatalab.org/shdi/shdi/.

[29] Zhao FH, Chen JF, Gao XH, Gao LM, Liu QG, Liu ZH, et al [Effectiveness and health economic analysis of strategies on cervical cancer screening and early diagnosis and treatment]. Zhonghua zhong liu za zhi [Chinese journal of oncology]. 2012;34(8):632–6. Epub 2012/11/20. 10.3760/cma.j.issn.0253-3766.2012.08.017 . 23159002

[30] Shenzhen Statistics Bureau. Shenzhen Statistical Yearbook 2018: China Statistics Press; 2018.

[31] Guangdong Statistics Bureau. Statistical Bulletin on Economic and Social Development of Guangdong in 2017. http://stats.gd.gov.cn/tjgb/content/post_1430134.html.

[32] Beijing Statistics Bureau. Beijing Statistical Yearbook 2018: China Statistics Press; 2018.

[33] Shi JF, Shi CL, Yue XP, Huang HY, Wang L, Li J, et al [Economic burden of cancer in China during 1996–2014: a systematic review]. Zhonghua zhong liu za zhi [Chinese journal of oncology]. 2016;38(12):929–41. Epub 2016/12/22. 10.3760/cma.j.issn.0253-3766.2016.12.010 . 27998471

[34] Deb S. Gap between GDP and HDI: Are the Rich Country Experiences Different from the Poor? IARIW-OECD Special Conference; Paris, France2015.

[35] Gao L, Hu H, Zhao FL, Li SC. Can the Direct Medical Cost of Chronic Disease Be Transferred across Different Countries? Using Cost-of-Illness Studies on Type 2 Diabetes, Epilepsy and Schizophrenia as Examples. PloS one. 2016;11(1):e0147169 Epub 2016/01/28. 10.1371/journal.pone.0147169 26814959 PMC4731392

[36] Meng Kai LD, Guan Zhongjun, Zhai Xiaohui. [Comparative study of medical service pricing of Beijing with those of others provinces]. Chin J Hosp Admin. 2016;32(2):84–8.

[37] Meng Q, Yang H, Chen W, Sun Q, Liu X. People’s Republic of China: health system review: World Health Organization; 2015.

[38] Toy M, Hutton DW, So SK. Cost-Effectiveness and Cost Thresholds of Generic and Brand Drugs in a National Chronic Hepatitis B Treatment Program in China. PloS one. 2015;10(11):e0139876 Epub 2015/11/05. 10.1371/journal.pone.0139876 26536626 PMC4633043

