# Supplementary material for: Development and application of a framework to estimate health care costs in China: The cervical cancer example
Source: PLoS One. 2019 Oct 1;14(10):e0222760. doi: 10.1371/journal.pone.0222760 (PMC6773209; doi:10.1371/journal.pone.0222760)
Supplement: S1 Fig — (PDF) [file pone.0222760.s001.pdf]

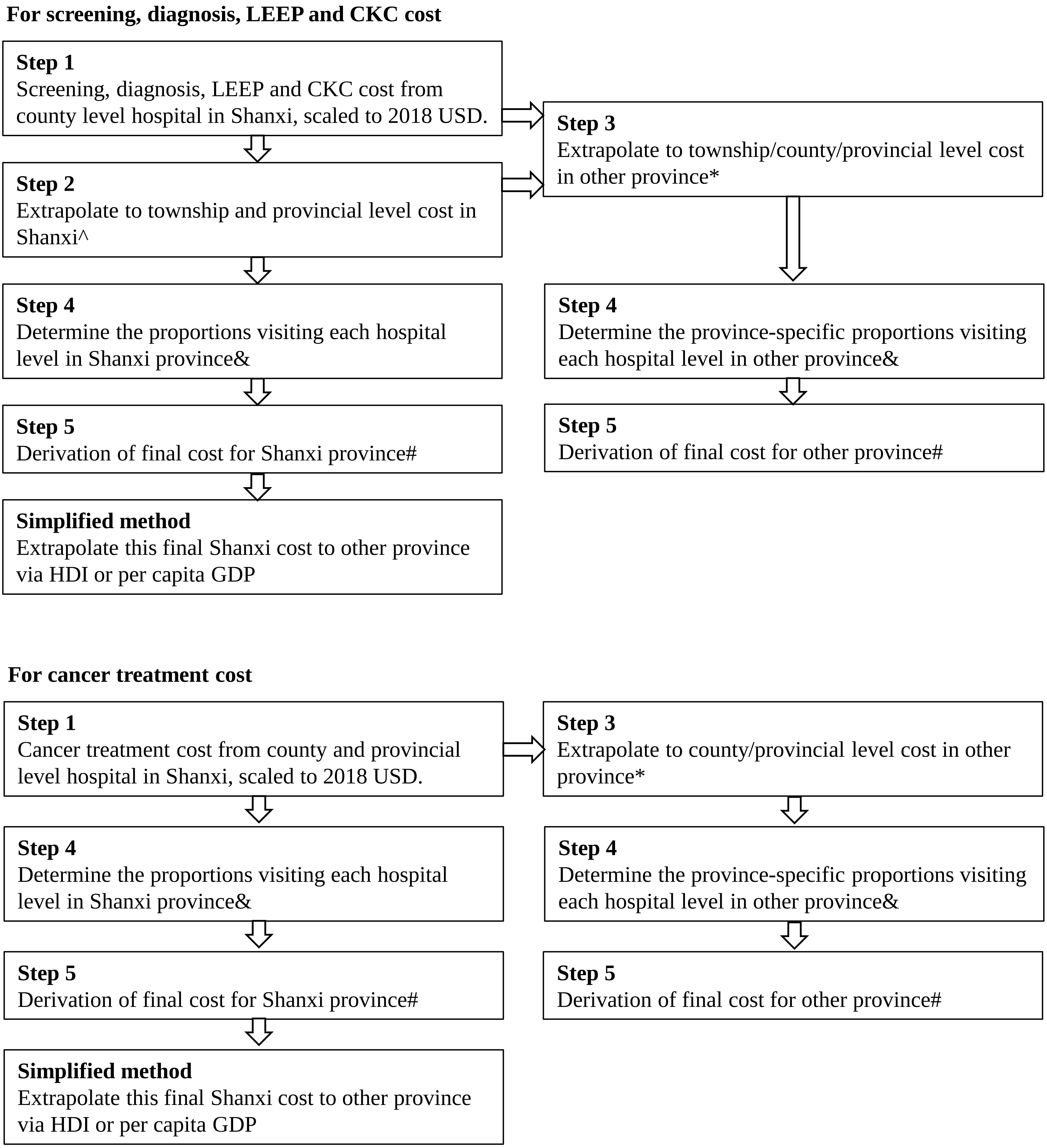

S1 Fig. The flow charts for the detailed method and the simplified method

Step 1-5 belongs to the detailed methods.

^ Extrapolate the original data at county-level hospitals to township/provincial hospital levels by applying separate multipliers to each component (supplies, equipment, staff, drugs, or programmatic), the multipliers shown in S3 Table and the specific process is shown in S2 Fig.

* Costs from Shanxi province were extrapolated to other provinces based on average provincial variations for both inpatient and outpatient costs. Outpatient costs were assumed to include screening, diagnostic procedures as well as LEEP; inpatient costs were assumed to include CKC and cancer treatment. The province-specific average inpatient and outpatient costs are shown in S5 Table and the specific process is shown in S2 Fig.

& The urban and rural proportions of the population along with the hospital-seeking behaviour of these urban and rural populations can be used to obtain proportions visiting each hospital level. The urban population proportions showed in S3 Fig and hospital-seeking behaviour described in S6 Table.

The resultant proportions for each province were multiplied by specific hospital level costs in each province to obtain final provincial weighted average cost for each procedure.
