# Supplementary material for: Development and application of a framework to estimate health care costs in China: The cervical cancer example
Source: PLoS One. 2019 Oct 1;14(10):e0222760. doi: 10.1371/journal.pone.0222760 (PMC6773209; doi:10.1371/journal.pone.0222760)
Supplement: S2 Fig — (PDF) [file pone.0222760.s002.pdf]

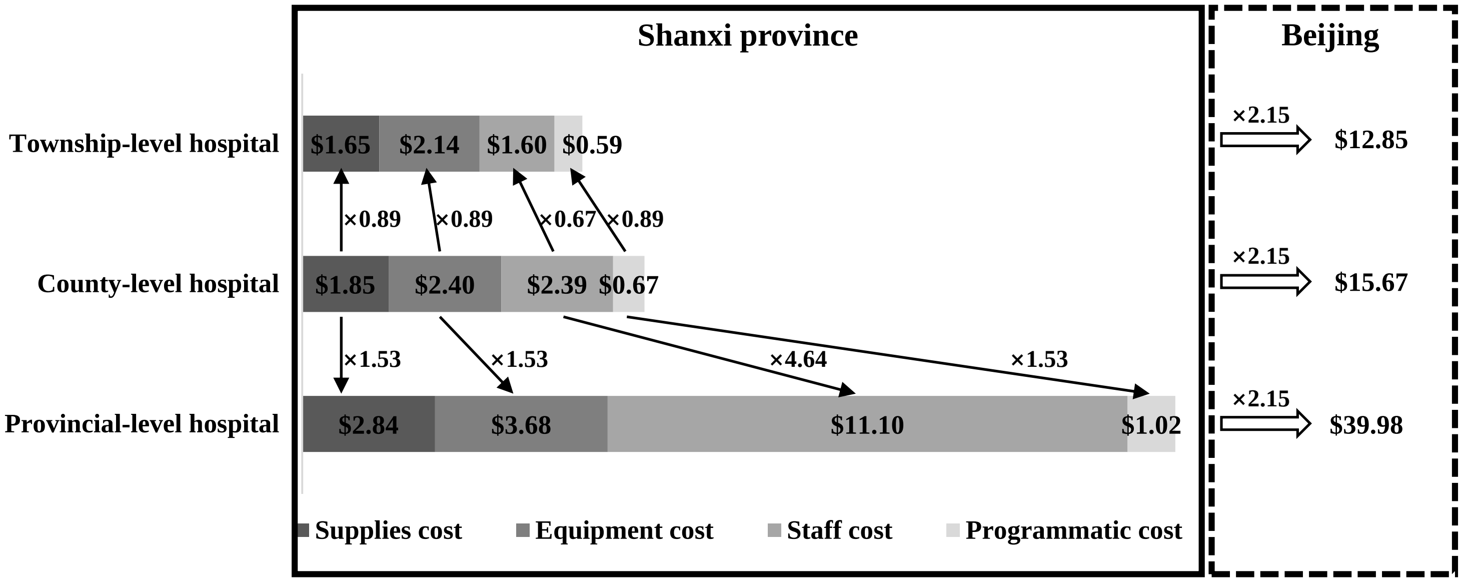

S2 Fig. The detail process of step 2-3 (using a specific example of biopsies)

Diagram in the solid line box showed how to extrapolate county level screening, diagnosis, LEEP and CKC cost (using a specific example of biopsies) to township and provincial levels in Shanxi province. Diagram in the dotted line box showed extrapolate these three hospital level cost in Shanxi to other province (using a specific example of Beijing).
