# Supplementary figures and images for: Development and application of a framework to estimate health care costs in China: The cervical cancer example
Source: PLoS One. 2019 Oct 1;14(10):e0222760. doi: 10.1371/journal.pone.0222760 (PMC6773209; doi:10.1371/journal.pone.0222760)

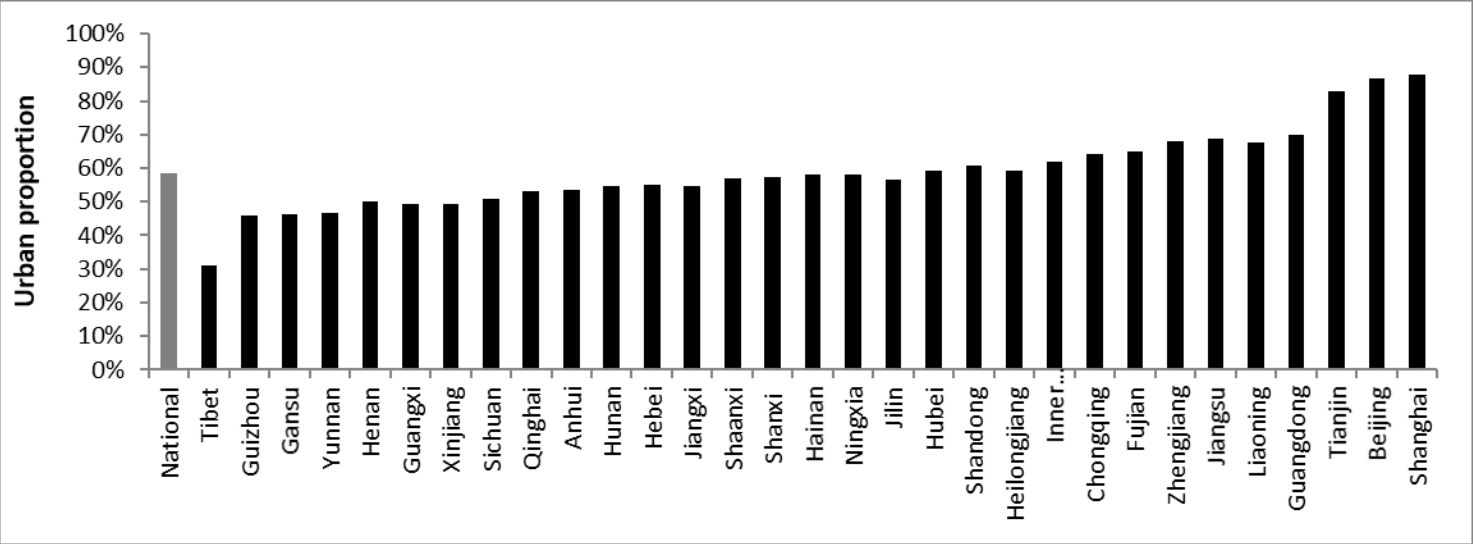

S3 Fig. The urban percentage of the total population by province

Supplement: S3 Fig — (PDF) [file pone.0222760.s003.pdf]

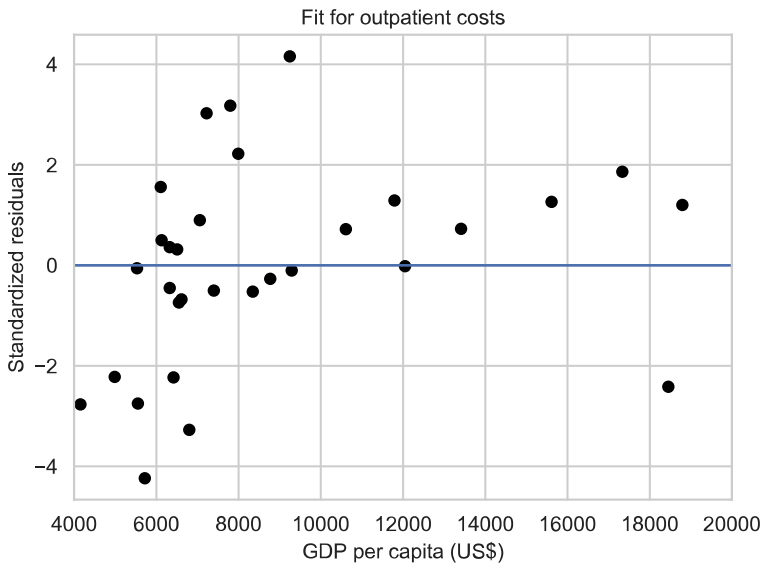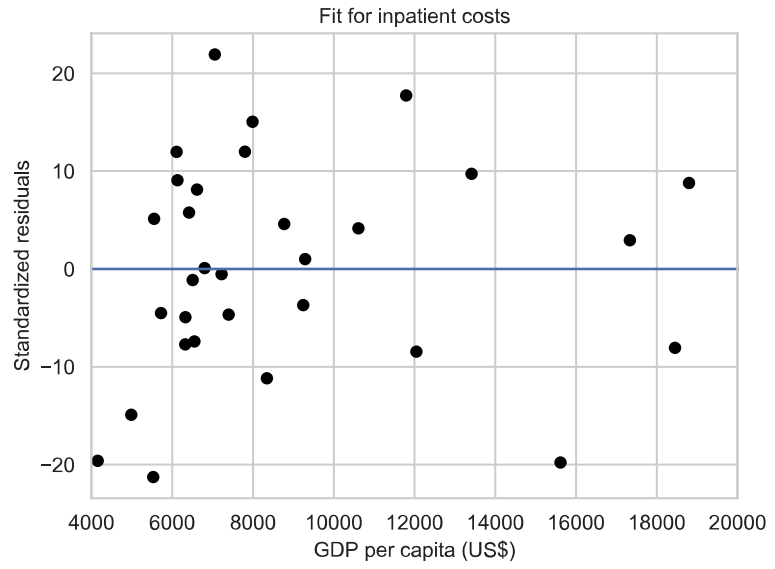

**S4 Fig. Residual plots of outpatient and inpatient costs for the GDP-based exponential fit**

Supplement: S4 Fig — (PDF) [file pone.0222760.s004.pdf]

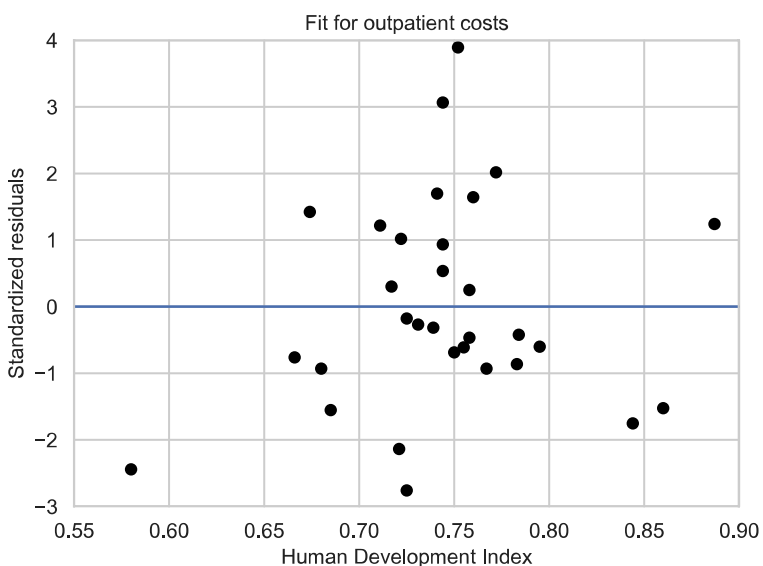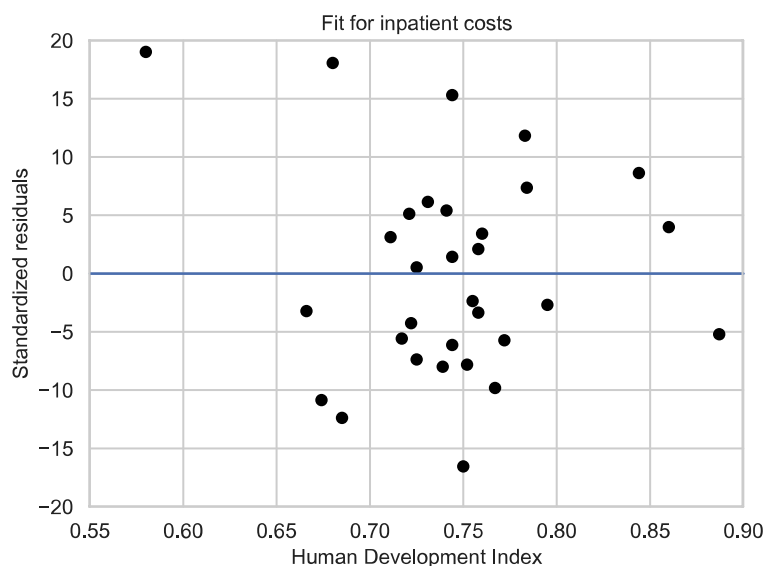

**S5 Fig. Residual plots of outpatient and inpatient costs for the HDI-based exponential fit**

Supplement: S5 Fig — (PDF) [file pone.0222760.s005.pdf]
