# Supplementary material for: Development and application of a framework to estimate health care costs in China: The cervical cancer example
Source: PLoS One. 2019 Oct 1;14(10):e0222760. doi: 10.1371/journal.pone.0222760 (PMC6773209; doi:10.1371/journal.pone.0222760)
Supplement: S1 Table — (DOCX) [file pone.0222760.s006.docx]

**S1 Table. Direct medical costs for cervical screening, diagnosis and pre-cancer treatment from micro-costing study (2018US$)**

|  | Supplies | Equipment | Staff | Drugs | Programmatic | Unit cost |
| --- | --- | --- | --- | --- | --- | --- |
| **Screening cost** |  |  |  |  |  |  |
| VIA | 0.38 | 0.004 | 0.82 | - | 1.63 | 2.84 |
| VIA/VILI | 0.43 | 0.004 | 1.19 | - | 1.72 | 3.35 |
| careHPV* (self-sampling) | 5.84 | 1.29 | 0.28 |  | 2.08 | 9.50 |
| careHPV* (clinician-sampling) | 6.17 | 1.29 | 0.86 |  | 1.76 | 10.08 |
| HC2^#^(self-sampling) | 8.00 | 0.78 | 0.48 |  | 2.08 | 11.34 |
| HC2^#^ (clinician-sampling) | 8.32 | 0.78 | 1.05 |  | 1.76 | 11.92 |
| **Diagnosis cost** |  |  |  |  |  |  |
| Colposcopy | 0.40 | 2.14 | 1.12 | - | 1.29 | 4.95 |
| Biopsy | 1.85 | 2.40 | 2.39 | - | 0.67 | 7.30 |
| ECC | 1.60 | 1.89 | 1.85 | - | 0.53 | 5.87 |
| **Pre-cancer treatment cost** |  |  |  |  |  |  |
| LEEP | 19.14 | 36.02 | 14.49 | 7.00 | - | 76.65 |
| CKC | 47.79 | 42.75 | 40.30 | 22.16 | - | 153.01 |

1 USD=6.8632 CNY（31 December 2018）
*The careHPV assay (Qiagen, Gaithersburg, MD, USA); ^#^ The Hybrid Capture 2 (HC2) assay (Qiagen, Germantown, MD).

VIA, visual inspection with acetic acid; VILI, visual inspection with lugol’s iodine; HPV, human papillomavirus; ECC, endocervical curettage; LEEP, loop electrosurgical excision procedure; CKC, cold knife conisation; FIGO, International Federation of Gynaecology and Obstetrics.
