# Supplementary material for: Development and application of a framework to estimate health care costs in China: The cervical cancer example
Source: PLoS One. 2019 Oct 1;14(10):e0222760. doi: 10.1371/journal.pone.0222760 (PMC6773209; doi:10.1371/journal.pone.0222760)
Supplement: S2 Table — (DOCX) [file pone.0222760.s007.docx]

**S2 Table. Direct medical costs for cervical cancer treatment from previous study (2018 US$)**

| Clinical diagnosis | FIGOⅠ | FIGOⅡ | FIGOⅢ | FIGOⅣ |
| --- | --- | --- | --- | --- |
| County level | 669.73 | 476.52 | 851.28 | 714.07 |
| Provincial level | 1703.43 | 2020.32 | 1967.70 | 1816.23 |

1 USD=6.8632 CNY（31 December 2018） FIGO: International Federation of Gynaecology and Obstetrics
