# Supplementary material for: Development and application of a framework to estimate health care costs in China: The cervical cancer example
Source: PLoS One. 2019 Oct 1;14(10):e0222760. doi: 10.1371/journal.pone.0222760 (PMC6773209; doi:10.1371/journal.pone.0222760)
Supplement: S3 Table — (DOCX) [file pone.0222760.s008.docx]

**S3 Table. Multipliers used to extrapolate county-level cost to township and provincial level in Shanxi province**

| Cost component | Inpatient cost | | | Outpatient cost | | |
| --- | --- | --- | --- | --- | --- | --- |
|  | Township | County | Provincial | Township | County | Provincial |
| Supplies | 0.59 | 1.00 | 3.24 | 0.89 | 1.00 | 1.53 |
| Equipment | 0.72 | 1.00 | 2.05 | 0.89 | 1.00 | 1.53 |
| Staff | 0.67 | 1.00 | 4.64 | 0.67 | 1.00 | 4.64 |
| Drugs | 0.72 | 1.00 | 2.09 | 0.85 | 1.00 | 1.65 |
| Programmatic | 0.72 | 1.00 | 2.05 | 0.89 | 1.00 | 1.53 |

The calculation process described further in the S4 Table.
