# Supplementary material for: Development and application of a framework to estimate health care costs in China: The cervical cancer example
Source: PLoS One. 2019 Oct 1;14(10):e0222760. doi: 10.1371/journal.pone.0222760 (PMC6773209; doi:10.1371/journal.pone.0222760)
Supplement: S4 Table — (DOCX) [file pone.0222760.s009.docx]

**S4 Table. National average cost by hospital level based on data from the 2018 China Health Statistics Yearbook (partial data) (2018 US$)**

|  |  | Provincial | Prefectural* | County | Township |
| --- | --- | --- | --- | --- | --- |
| 1 | Average hospital expenditure on staff wage | 18107588 | 200090920 | 74962991 | 29376384 |
| 2 | Average outpatient cost per visit | 37.50 | 69.37 | 52.79 | 38.95 |
| 3 | *Drug* | 15.55 | 32.16 | 22.90 | 16.29 |
| 4 | Average inpatient cost per person | 1418.49 | 3347.90 | 2562.64 | 1689.43 |
| 5 | *Supplies* | 279.85 | 990.91 | 667.43 | 324.59 |
| 6 | *Drug* | 435.09 | 996.21 | 802.24 | 516.77 |

1 USD=6.8632 CNY（31 December 2018）
* Prefectural level is contained in provincial level, so the average of these two is taken as provincial level value.
The first row data was used to calculate the staff wages differentials between hospitals. Row 3 and 6 were used to calculate drugs multipliers for outpatient and inpatient services, respectively. Row 5 was used for supply multipliers in inpatient service. Row 2 and 4 were taken as average differentials for outpatient and inpatient service and used to calculate supply in outpatient service and equipment, programmatic for both outpatient and inpatient service.
