# Supplementary material for: Development and application of a framework to estimate health care costs in China: The cervical cancer example
Source: PLoS One. 2019 Oct 1;14(10):e0222760. doi: 10.1371/journal.pone.0222760 (PMC6773209; doi:10.1371/journal.pone.0222760)
Supplement: S5 Table — (DOCX) [file pone.0222760.s010.docx]

**S5 Table. Province-specific hospital outpatient visit costs and hospital inpatient bed day costs (2018 US$)**

| Region | Outpatient visit costs | | Inpatient bed day costs | |
| --- | --- | --- | --- | --- |
|  | Cost | Ratio to Shanxi | Cost | Ratio to Shanxi |
| Beijing | 75.30 | 2.15 | 3167.21 | 2.58 |
| Shanghai | 52.60 | 1.50 | 2649.67 | 2.16 |
| Tianjin | 46.16 | 1.32 | 2408.02 | 1.96 |
| Chongqing | 43.23 | 1.23 | 1148.90 | 0.93 |
| Liaoning | 41.04 | 1.17 | 1305.24 | 1.06 |
| Hunan | 40.71 | 1.16 | 1128.96 | 0.92 |
| Jilin | 38.92 | 1.11 | 1350.73 | 1.10 |
| Jiangsu | 38.00 | 1.08 | 1544.16 | 1.26 |
| Heilongjiang | 37.45 | 1.07 | 1266.07 | 1.03 |
| Guangdong | 36.86 | 1.05 | 1591.44 | 1.29 |
| Hainan | 35.97 | 1.03 | 1413.54 | 1.15 |
| Zhejiang | 35.70 | 1.02 | 1647.98 | 1.34 |
| Shandong | 35.58 | 1.01 | 1319.66 | 1.07 |
| **Shanxi** | **35.09** | **1.00** | **1229.41** | **1.00** |
| Jiangxi | 34.78 | 0.99 | 1054.55 | 0.86 |
| Sichuan | 34.68 | 0.99 | 1106.26 | 0.90 |
| Fujian | 33.96 | 0.97 | 1244.99 | 1.01 |
| Guizhou | 33.85 | 0.96 | 834.58 | 0.68 |
| Inner Mongolia | 33.75 | 0.96 | 1212.90 | 0.99 |
| Hubei | 33.38 | 0.95 | 1240.34 | 1.01 |
| Anhui | 32.97 | 0.94 | 1018.96 | 0.83 |
| Ningxia | 32.86 | 0.94 | 1079.69 | 0.88 |
| Shaanxi | 32.81 | 0.94 | 1020.38 | 0.83 |
| Hebei | 32.46 | 0.93 | 1226.51 | 1.00 |
| Xinjiang | 32.33 | 0.92 | 1026.75 | 0.84 |
| Yunnan | 29.04 | 0.83 | 908.47 | 0.74 |
| Qinghai | 29.01 | 0.83 | 1192.67 | 0.97 |
| Guangxi | 27.86 | 0.79 | 1169.73 | 0.95 |
| Gansu | 27.82 | 0.79 | 840.69 | 0.68 |
| Henan | 26.69 | 0.76 | 1127.74 | 0.92 |
| Tibet | 24.55 | 0.70 | 1049.95 | 0.85 |

1 USD=6.8632 CNY（31 December 2018）. Sorted by outpatient costs in descending order.
