# Supplementary material for: Development and application of a framework to estimate health care costs in China: The cervical cancer example
Source: PLoS One. 2019 Oct 1;14(10):e0222760. doi: 10.1371/journal.pone.0222760 (PMC6773209; doi:10.1371/journal.pone.0222760)
Supplement: S6 Table — (DOCX) [file pone.0222760.s011.docx]

**S6 Table. Hospital-seeking behaviour by urban/rural and region (%)**

| Hospital level | Urban | | | | Rural | | | |
| --- | --- | --- | --- | --- | --- | --- | --- | --- |
|  | Urban average | Eastern | Middle | Western | Rural average | Eastern | Middle | Western |
| *Outpatient** |  |  |  |  |  |  |  |  |
| Township | 67.4 | 64.1 | 66.5 | 71.5 | 81.2 | 81.8 | 82.9 | 78.8 |
| County | 18.2 | 19.4 | 21.5 | 14.5 | 16.3 | 15.9 | 15.0 | 181 |
| Provincial | 14.5 | 16.5 | 12.0 | 14.0 | 2.5 | 2.2 | 2.1 | 3.1 |
| Total | 100 | 100 | 100 | 100 | 100 | 100 | 100 | 100 |
| *Inpatient&* |  |  |  |  |  |  |  |  |
| County | 55.4 | 54.6 | 53.1 | 58.1 | 81.2 | 79.9 | 82.2 | 81.1 |
| Provincial | 44.6 | 45.4 | 46.9 | 41.9 | 18.8 | 20.1 | 17.8 | 18.9 |
| Total | 100 | 100 | 100 | 100 | 100 | 100 | 100 | 100 |

*Proportions estimated from the total of township/county/provincial (i.e. not including the “other” category).
& Proportions estimated from the total of county/provincial (i.e. not including the “other” category).
Percentages are based on first contact or first visit for a given disease.
Eastern region includes 11 provinces:- Beijing, Tianjin, Hebei, Liaoning, Jiangsu, Shanghai, Zhejiang, Fujian, Guangdong, Hainan, Shandong; Middle region includes 8 provinces: - Jilin, Heilongjiang, Shanxi, Henan, Anhui, Hubei, Hunan, Jiangxi; Western region includes 12 provinces:- Innter Mongolia, Guangxi, Chongqing, Sichuan, Guizhou, Yunnan, Tibet, Shaanxi, Gansu, Qinghai, Ningxia, Xinjiang.
