# Supplementary material for: Development and application of a framework to estimate health care costs in China: The cervical cancer example
Source: PLoS One. 2019 Oct 1;14(10):e0222760. doi: 10.1371/journal.pone.0222760 (PMC6773209; doi:10.1371/journal.pone.0222760)
Supplement: S8 Table — (DOCX) [file pone.0222760.s013.docx]

**S8 Table. Percentage difference of various fitting functions and data**

| **Parameter 1 (x_1_)** | **Parameter 2 (x_2_)** | **Fitting function** | **Outpatient % average difference from detailed method** | **Inpatient % average difference from detailed method** |  |
| --- | --- | --- | --- | --- | --- |
| GDP | None | $y=A\exp(Bx_{1})$ | 13.9% | 20.0% |  |
| HDI | None | $y=A\exp(Bx_{1})$ | 8.3% | 11.5% |  |
| GDP | None | $y=A\exp(Bx_{1})+C$ | 11.3% | 15.8% |  |
| HDI | None | $y=A\exp(Bx_{1})+C$ | 8.1% | 9.3% |  |
| Household consumption | None | $y=A\exp(Bx_{1})+C$ | 11.5% | 16.0% |  |
| Doctors per capita | None | $y=A\exp(Bx_{1})+C$ | 12.6% | 15.5% |  |
| Hospital beds per capita | None | $y=A\exp(Bx_{1})+C$ | 14.6% | 20.9% |  |
| Proportion of primary hospitals | None | $y=A\exp(Bx_{1})+C$ | 14.6% | 21.4% |  |
| Proportion of secondary hospitals | None | $y=A\exp(Bx_{1})+C$ | 14.1% | 19.4% |  |
| Proportion of tertiary hospitals | None | $y=A\exp(Bx_{1})+C$ | 13.1% | 27.4% |  |
| GDP | HDI | $y=A\exp\left( Bx_{1}+Cx_{2} \right)+D$ | 8.5% | 10.3% |  |
| GDP | Household consumption | $y=A\exp\left( Bx_{1}+Cx_{2} \right)+D$ | 10.9% | 15.8% |  |
| GDP | Doctors per capita | $y=A\exp\left( Bx_{1}+Cx_{2} \right)+D$ | 10.5% | 15.9% |  |
| GDP | Hospital beds per capita | $y=A\exp\left( Bx_{1}+Cx_{2} \right)+D$ | 10.9% | 15.4% |  |
| GDP | Proportion of primary hospitals | $y=A\exp\left( Bx_{1}+Cx_{2} \right)+D$ | 10.6% | 15.7% |  |
| GDP | Proportion of secondary hospitals | $y=A\exp\left( Bx_{1}+Cx_{2} \right)+D$ | 10.5% | 15.2% |  |
| GDP | Proportion of tertiary hospitals | $y=A\exp\left( Bx_{1}+Cx_{2} \right)+D$ | 10.3% | 16.7% |  |
| HDI | Household consumption | $y=A\exp\left( Bx_{1}+Cx_{2} \right)+D$ | 7.8% | 9.5% |  |
| HDI | Doctors per capita | $y=A\exp\left( Bx_{1}+Cx_{2} \right)+D$ | 7.7% | 9.5% |  |
| HDI | Hospital beds per capita | $y=A\exp\left( Bx_{1}+Cx_{2} \right)+D$ | 8.0% | 8.5% |  |
| HDI | Proportion of primary hospitals | $y=A\exp\left( Bx_{1}+Cx_{2} \right)+D$ | 8.1% | 9.3% |  |
| HDI | Proportion of secondary hospitals | $y=A\exp\left( Bx_{1}+Cx_{2} \right)+D$ | 7.7% | 9.1% |  |
| HDI | Proportion of tertiary hospitals | $y=A\exp\left( Bx_{1}+Cx_{2} \right)+D$ | 7.8% | 10.0% |  |
